# Effects of arm and leg positions on lumbar multifidus muscle activity while on hands and knees or while standing

**DOI:** 10.1186/s40101-022-00279-z

**Published:** 2022-03-07

**Authors:** Yasuhiro Mitani, Masaru Hanafusa, Junichi Hashimoto, Ryuta Inada, Hitoshi Koda

**Affiliations:** 1 Department of Rehabilitation Sciences, Faculty of Allied Health, Kansai University of Welfare Sciences, 3-11-1 Asahigaoka, Kashiwara, Osaka 582-0026 Japan; 2 An Visiting Nursing Station, 1-49-28 Kamikamida, Neyagawa, Osaka 572-0052 Japan; 3 Graduate School of Comprehensive Rehabilitation, Osaka Prefecture University, 3-7-30 Habikino, Habikino, Osaka 583-8555 Japan; 4 Department of Rehabilitation, Shimada Hospital, 100-1 Kashiyama, Habikino, Osaka 583-0875 Japan

**Keywords:** Electromyography, Lumbar spine, Low back pain

## Abstract

**Background:**

The lumbar multifidus (LMF) muscle, which is involved in the mechanical stability of the lumbar spine, reportedly undergoes atrophy in patients with low back pain. Preventing or mitigating low back pain requires strengthening the LMF muscle; however, methods for triggering selective and significant contraction of this muscle have not been fully studied. This study aims to clarify how, in the hands-and-knees or standing position, the position of the arm and leg on one side affects the activity of the lumbar erector spinae (LES) and LMF muscles.

**Methods:**

We recruited nine adult men with no prior history of low back pain. Measurements were taken in four different postures under varying conditions (that is, one arm and one leg were lifted in either the hands-and-knees or standing position,) as follows: (1) shoulder joint flexion and hip joint extension in the hands-and-knees position; (2) 90° shoulder joint abduction and hip joint abduction in the hands-and-knees position; (3) shoulder joint flexion and hip joint extension in the standing position; and (4) 90° shoulder joint abduction and hip joint abduction in the standing position. The 90° shoulder joint abduction involved simultaneous horizontal abduction, while the hip joint abduction involved simultaneous extension. Muscle activity of the LES and LMF in each posture was measured using a surface electromyograph.

**Results:**

Muscle activity of the LMF was significantly higher in 90° shoulder joint abduction and hip joint abduction than in shoulder joint flexion and hip joint extension in both the hands-and-knees and standing positions. The LES muscle showed no significant differences in activity between each posture.

**Conclusions:**

The results suggest that unilateral 90° shoulder joint abduction and contralateral hip joint abduction in the hands-and-knees and standing positions may produce selective and significant contraction of the LMF muscle.

## Background

Lumbar instability is also one of the causes of low back pain [1, 2], and lumbar multifidus (LMF) muscle function is particularly important [3]. The LMF reportedly atrophies in patients with low back pain (LBP) [4–6]. LBP reduces the ability to stand and walk, thus limiting activities of daily living [7, 8]. Therefore, strengthening the LMF muscle may help stabilize the lumbar spine, alleviate LBP, and improve related standing and walking abilities. Common methods of strengthening the LMF muscle include the back bridge exercise and lifting one arm and contralateral leg while on hands and knees [9]. Okubo et al. [10] also reported that the back bridge exercise increased LMF muscle activity and that lifting one leg further increased the muscle’s activity. However, these methods increase not only the muscle activity of the LMF but also that of the lumbar erector spinae (LES) [9, 10]. Masaki et al. [11] reported that lifting one arm and leg while on hands and knees, with 90° shoulder and contralateral hip joint abduction, produced selective contraction of the LMF muscle on the side of the abducted hip. The LMF muscle had low activity at the time of this study; however, this may have been because the load was not sufficient to increase muscle strength.

Because being on hands and knees involves hip flexion, the pelvis is tilted backward, and there is limited lumbar lordosis associated with pelvic anteversion, even with one arm and one leg lifted. Consequently, an adequate load may not be applied to the LMF muscle. Therefore, we propose a method in which one arm and a contralateral leg are lifted in the standing position, with the hand placed on a bed in front of the patient and mild anteversion of the trunk. This method involves a small angle degree of flexion of the hip joint; when one arm and a contralateral leg are lifted, pelvic anteversion and lumbar lordosis are not restricted, and LMF muscle activity is increased. However, LMF muscle activity in this posture has not been adequately studied.

Exercises for strengthening the LMF muscle have been performed in the supine and hands-and-knees positions, but never in the standing position. Methods for strengthening the LMF muscle in the standing position have not been adequately investigated. If exercises for strengthening the LMF muscle can be performed in the standing position, they will be more efficient and are likely to yield excellent results. Therefore, this study aims to clarify how, in the hands-and-knees or standing position, the position of the arm and leg on one side affects the muscle activity of the LES and LMF, and to study methods for effectively strengthening the LMF.

## Methods

### Participants

We recruited nine adult men with no prior history of LBP and a mean age ± standard deviation [SD] of 25.0 ± 6.7 years; height, 171.7 ± 5.2 cm; and body weight, 62.4 ± 6.3 kg. The study objectives and methods were explained to all participants, who then gave their consent to participate in the study. This study was approved by the Research Ethics Committee of Kansai University of Welfare Sciences (approval number: 20–23).

### Measurements

**Fig. 1:**
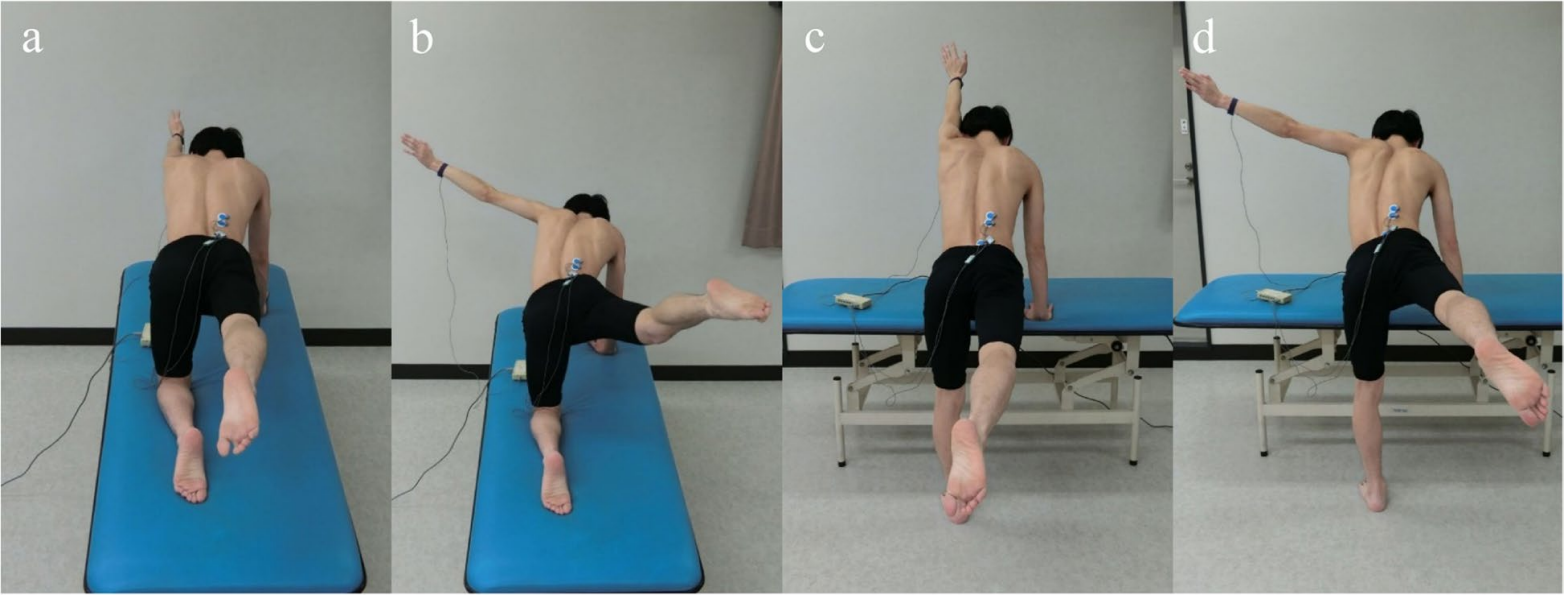
Measurement postures. **a** Shoulder flexion/hip extension in the hands and knees position (SFHE-HK). **b** 90° shoulder abduction/hip abduction in the hands and knees position (SAHA-HK). **c** Shoulder flexion/hip extension in the standing position (SFHE-S). **d** 90° shoulder abduction/hip abduction in the standing position (SAHA-S)

Measurements were taken in four different postures in which the arm of the non-dominant hand and the contralateral leg were lifted in either the hands-and-knees or standing position. The different postures were as follows: (1) shoulder flexion and hip extension in the hands-and-knees position (SFHE-HK) (Fig. 1a); (2) 90° shoulder abduction and hip abduction in the hands-and-knees position (SAHA-HK) (Fig. 1b); (3) shoulder flexion and hip extension in the standing position (SFHE-S) (Fig. 1c); and (4) 90° shoulder abduction and hip abduction in the standing position (SAHA-S) (Fig. 1d). In the standing position, one hand was placed on a vertical lifting bed that had been placed in front of the participant, and the trunk was anteverted 45°. SFHE-HK and SFHE-S had maximum flexion of the shoulder joint and maximum extension of the hip joint. SAHA-HK and SAHA-S had the shoulder joint at maximum horizontal abduction simultaneously with 90° abduction and the hip joint at maximum extension simultaneously with maximum abduction. In all cases, the elbow and knee joints were extended and lumbar lordosis, lateral flexion, and rotation were not restricted.

A surface electromyograph (SX230FW, Biometrics Ltd., Newport, UK) was used for electromyography. The sampling frequency was 1000 Hz. The muscles tested were the LES and the LMF on the side of the abducted leg. Electromyograph electrodes were affixed 3 cm lateral from the spinous process of the second and fourth lumbar vertebra for the LES and LMF muscles, respectively [12]. The skin was fully treated before electrode placement to reduce the contact resistance between the electrodes and the skin. The maximum strength (maximum voluntary contraction [MVC]) of each muscle under test was measured in advance, for 5 s, and the mean amplitude spanning the first to fourth seconds was determined. Muscle activity was measured for 5 s in each posture, the mean amplitude for the first to fourth seconds was determined, and the proportion (%MVC) relative to muscle activity in MVC was calculated. Measurements were taken twice in each posture, and the mean value was calculated. The order in which measurements in the various postures were taken was random, with 5 min of rest between measurements to avoid muscle fatigue. Data processing for the surface electromyograph was performed using analysis software (TRIAS, DKH). The raw waveform of the electromyograph was processed with a band-pass filter (10–450 Hz), followed by full-wave rectification and smoothing with a low-pass filter (20 Hz).

### Statistics analyses

Data are presented as mean ± SD. Statistical analysis was performed using IBM SPSS Statistics for Windows, version 24 (IBM Corp., Armonk, NY, USA). The normality of distribution of the data was confirmed using the Shapiro-Wilk test. A repeated measures analysis of variance was performed, followed by Tukey’s post hoc test for multiple comparisons. The level of significance was set at *P* < 0.05.

## Results

Table 1 shows the LES and LMF muscle activity in each posture. The LMF muscle activity was 38.7 ± 11.0 %MVC in SFHE-HK, 53.1 ± 17.1 %MVC in SAHA-HK, 34.8 ± 16.7 %MVC in SFHE-S, and 53.4 ± 13.1 %MVC in SAHA-S, with SAHA-HK showing significantly higher values than SFHE-HK (*P* = 0.027, *r* = 0.616) and SFHE-S (*P* = 0.004, *r* = 0.795). SAHA-S also had significantly higher values than SFHE-HK (*P*= 0.024, *r* = 0.757) and SFHE-S (*P* = 0.003, *r* = 0.882); the LES muscle showed no significant differences in activity between these postures.

**Table 1:** LES and LMF muscle activity in each posture

		SFHE-HK	SAHA-HK	SFHE-S	SAHA-S	*P*-value	η^2^
LES	%MVC (mean ± SD)	33.1 ± 12.5	38.3 ± 8.9	30.3 ± 8.2	41.1 ± 8.5	*P =* 0.120	0.258
	95% CI	23.5 to 42.7	31.5 to 45.1	24.1 to 36.6	34.5 to 47.6		
LMF	%MVC (mean ± SD)	38.7 ± 11.0	53.1 ± 17.1^a,b^	34.8 ± 16.7	53.4 ± 13.1^c,d^	*P =* 0.001**	0.510
	95% CI	30.3 to 47.1	39.9 to 66.2	22.0 to 47.7	43.3 to 63.4		

^a^ SAHA-HK exhibited significantly higher values than SFHE-HK (*P* = 0.027*, *r* = 0.616), ^b^ SAHA-HK exhibited significantly higher values than SFHE-S (*P* = 0.004**, *r* = 0.795), ^c^ SAHA-S exhibited significantly higher values than SFHE-HK (*P* = 0.024*, *r* = 0.757), ^d^ SAHA-S exhibited significantly higher values than SFHE-S (*P* = 0.003**, *r* = 0.882). *P < .05, **P < .01, with statistical significance. η^2^, r: effect size, *MVC* maximum voluntary contraction. *CI* confidence interval

## Discussion

The methods for strengthening the LMF muscle commonly involve the participant positioned on their hands and knees, with one of the shoulder joints flexed and the contralateral hip joint extended. However, muscle activity in this posture was reported to be 30 %MVC (low) by Callaghan et al. [13] and 28.5 ± 10.0 %MVC by Masaki et al. [11]. In the present study, the LMF muscle activity was around 30 %MVC in SFHE-HK, which was SFHE-S. However, SAHA-HK and SAHA-S, which involve hip and shoulder abduction, had significantly higher LMF muscle activity, probably because keeping the arm lifted and the leg in the abducted position produced rotational momentum in the spine due to their weight. The activity of the LMF muscle, which acts to rotate the lumbar spine, could have increased in order to resist this momentum. Masaki et al. [11] reported that with one shoulder joint in 90° abduction and the contralateral hip joint abducted in the hands-and-knees position, LMF muscle activity was around 30 %MVC, but SAHA-HK and SAHA-S in the present study yielded greater muscle activity at around 50 %MVC. Unlike Masaki et al.’s method [11], the present study involved simultaneous horizontal abduction and 90° abduction of the shoulder joint and extension and abduction of the hip joint, which caused lordosis, lateral flexion, and rotation of the lumbar spine in both the hands-and-knees and standing positions, resulting in greater muscle activity of the LMF, which is involved in all of these actions [14]. Neither SAHA-HK nor SAHA-S exhibited high values for LES muscle activity; there was no significant difference between these postures. Because the LES antagonizes the rotatory action of the LMF muscle, the activity promoted by 90° shoulder joint abduction and hip joint abduction is that of the LMF muscle on the side of the abducted hip joint. There was no increase in ipsilateral LES muscle activity in this posture. Isometric contraction for muscle strengthening is thought to require a load of 40–50 %MVC [15]. SFHE-HK and SFHE-S in the present study had a LES and LMF muscle activity ≤ 40 %MVC, which is inadequate for muscle strengthening. SAHA-HK and SAHA-S also had a LES muscle activity ≤ 40% but had LMF muscle activity at around 50 %MVC, which has the potential for selectively strengthening the LMF.

Queiroz et al. [16] reported that LMF muscle activity is increased in the pelvic anteversion with the extended trunk position. Beneck et al. [17] measured LMF muscle activity in trunk stabilization exercises using a variable-angle Roman chair and reported that LMF muscle activity increased in the trunk anteversion position. Therefore, it may not be necessary to lift the upper and lower limbs while in the trunk anteversion position, since the position alone increases LMF muscle activity. However, if the LMF on one side is atrophied in patients with LBP, it may be compensated for by the activity of the contralateral LMF muscle. SAHA-HK and SAHA-S in this study increased the activity of the LMF muscle on the side of the abducted leg, which may be useful as a method of selectively strengthening the LMF when it is atrophied on one side. Patients with unilateral LBP reportedly have a reduced cross-sectional area of the LMF muscle on the affected side [18, 19]. Preventing or mitigating LBP therefore requires strengthening of the LMF muscle, suggesting that SAHA-HK and SAHA-S may be useful in doing so.

In the present study, there was no significant difference in LES and LMF muscle activity between the hands-and-knees and standing positions. The upper body weight was supported by one arm in both the hands-and-knees and standing positions. However, because the lifted arm and leg are affected by gravity, the activity of LES and LMF muscles is likely to increase in the hands-and-knees position compared to the standing position. In contrast, pelvic anteversion and lumbar lordosis decrease with hip flexion [20, 21], and are therefore, likely to be associated with lower muscle activity in the hands-and-knees position, which is associated with a larger hip flexion angle than the standing position. Since the LES and LMF muscles both have an effect on lumbar spine extension, their activity is suppressed in the hands-and-knees position, where lumbar lordosis is reduced compared to the standing position. Therefore, the activity of the LES and LMF muscles is affected by gravity (affecting the lifted arm and leg), pelvic anteversion, and lumbar lordosis. We attributed the lack of difference in the LES and LMF muscle activity to a combination of these factors.

The trunk muscles are classified into local and global systems based on their function [22]. The large muscles of the superficial trunk make up the global system. They are involved in larger movements and exert force throughout the spinal column. The small, deeper layers comprise the local system that forms the lumbar curvature and provides mechanical stability between vertebral bodies. The interaction between these systems enables stable spinal movements. We found that LES muscle activity was not significantly increased in the various postures. However, LMF muscle activity was increased in SAHA-HK and SAHA-S. Therefore, even during lordosis, lateral flexion, and rotation of the lumbar spine in SAHA-HK and SAHA-S, the LMF muscle activity in the local system is considered to provide stability between the vertebrae.

In this study, LMF muscle activity was measured using surface electromyography. It has been reported that LMF muscle activity has a high correlation with surface and intramuscular electromyography [23]. Further, it has also been reported that peak electromyography amplitudes of the deep muscle (the LMF) are smaller on the surface than in the intramuscular electromyography [23]. In addition, it has been reported that the surface electromyography of the LMF is mixed in the myoelectric potentials of the longissimus muscle [23, 24]. Therefore, LMF muscle activity may have been influenced by the subcutaneous tissue and crosstalk of adjacent muscles in this study. It is necessary to measure LMF muscle activity using intramuscular electromyography in the future.

This study has several limitations. First, only healthy men were studied; therefore, the features of muscle activity in patients with LBP remain unknown. Patients with LBP need to be studied in the future. Moreover, the angles of the shoulder and hip joints, pelvis, and lumbar spine in each posture were not strictly set. SFHE-HK and SFHE-S had maximum flexion of the shoulder joint and maximum extension of the hip joint. SAHA-HK and SAHA-S had shoulder joint at maximum horizontal abduction with simultaneous 90° abduction and the hip joint at maximum extension with simultaneous maximum abduction. These are thought to have caused the lumbar spine to undergo lordosis, lateral flexion, and rotation, resulting in greater LMF muscle activity. It is necessary to clarify the angles of the shoulder and hip joints, pelvis, and lumbar spine in each posture and investigate the relationship between these angles and the lumbar muscle activity. However, even if each posture is unified when the range of motion of the shoulder or hip joint on the lifted side is decreased, it may be compensated for by an increase in the lumbar spine lordosis and lateral flexion. Compensation by the lumbar spine motion changes the LMF muscle activity. Beneck et al. [17] reported that instruction to increase lumbar spine lordosis in each posture of trunk stabilization exercises increased LMF muscle activity. Therefore, even if the same posture is held, the characteristics of LMF muscle activity may differ depending on the degree of compensation by the lumbar spine motion. Although the range of motion of the shoulder and hip joints, pelvis, and lumbar spine was not measured in this study, the range of motion of these joints and the degree of compensation at the lumbar spine in each posture may differ among participants. In interpreting the results of this study, the fact that the effect of each posture on LMF muscle activity may differ among participants must be taken into consideration, and it should be noted that comparing the measurement results depending on participants is not sufficiently valid.

## Conclusion

In both the hands-and-knees and standing positions, our results suggested that horizontal abduction with simultaneous 90° abduction of one shoulder joint and simultaneous extension with abduction of the contralateral hip joint is a useful method for achieving selective, significant contraction of the LMF muscle. The posture proposed in this study may be prescribed to strengthen LMF muscle activity and help prevent or alleviate LBP.

## Data Availability

The datasets used and/or analyzed during the current study are available from the corresponding author on reasonable request.

## Abbreviations

LMF: Lumbar multifidus muscle
LES: Lumbar erector spinae
SFHE-HK: Shoulder flexion and hip extension in the hands-and-knees position
SAHA-HK: 90° shoulder abduction and hip abduction in the hands-and-knees position
SFHE-S: Shoulder flexion and hip extension in the standing position
SAHA-S: 90° shoulder abduction and hip abduction in the standing position
